# Impetigo Herpetiformis Complicating Pregnancy: A Case Report on a Rare Gestational Dermatosis With Constitutional Symptoms

**DOI:** 10.7759/cureus.47898

**Published:** 2023-10-29

**Authors:** Ketav S Joshi, Shazia Mohammad, Neema Acharya, Samir Joshi

**Affiliations:** 1 Obstetrics and Gynecology, Jawaharlal Nehru Medical College, Datta Meghe Institute of Higher Education and Research, Wardha, IND; 2 Obstetrics and Gynecology, Dr. Vinod Joshi Maternity Nursing Home, Mumbai, IND

**Keywords:** pregnancy, prednisolone, cyclosporine, generalised pustular psoriasis, impetigo herpetiformis

## Abstract

Impetigo herpetiformis (IH) is a rare dermatosis that can manifest during the last trimester of pregnancy. It has the potential to cause fatality to both the mother and the fetus. After birth, it often vanishes spontaneously and rapidly. Clinically and histologically, it resembles pustular psoriasis, leading some authors to call it "the pustular psoriasis of pregnancy." Steroids were previously the treatment of choice, but treatment remains challenging. A dermatologist with experience in skin conditions during pregnancy should assess any generalized pustular psoriasis instances. There is a danger of stillbirth when a systemic sickness develops, so both the mother and fetus should be properly watched. A well-known side effect of pregnancy-related generalized pustular psoriasis is maternal sepsis. We report our own experience with a case of a 26-year-old pregnant woman who presented with IH that resolved postpartum.

## Introduction

During pregnancy, skin changes can be classified into three categories: physiological changes, pregnancy-related dermatoses, and other common skin problems. Dermatoses of pregnancy are a subset of skin conditions that occur only during pregnancy and the postpartum period. They include pemphigoid gestationis, atopic eruptions of pregnancy, and polymorphic eruption of pregnancy (PEP) [1]. A prevalent disease known as pemphigoid gestationis during the third trimester of pregnancy can result in severe itching, urticarial papules and plaques, and eventually bullae. Pruritic eruption of pregnancy (PEP), another disorder that causes itching, is more common towards the end of pregnancy and after giving birth. Atopic pregnancy eruptions can include eczema, prurigo, and pruritic folliculitis, according to Vaughan JS et al. [1]. There is a skin condition called generalized pustular psoriasis that causes inflammation. It is characterized by red patches with pustules on the skin, but it usually does not affect the face or extremities. When this skin condition appears during pregnancy, it is called generalized pustular psoriasis of pregnancy (GPPP), also known as impetigo herpetiformis (IH). This condition is different from other skin issues during pregnancy because it can cause fever, fatigue, elevated C-reactive protein, and leukocytosis. Doctors may need to do skin cultures to check for secondary infections, and a skin biopsy may be necessary. If the rash is severe, it can lead to erythrodermic skin, which can cause a loss of fluids and electrolytes, difficulty regulating body temperature, and other complications like sepsis and other infections [2].

A systemic disease in the mother increases the chance of fetal abnormalities, placental insufficiency, and stillbirth, necessitating thorough monitoring of the fetus [3,4]. A rare skin disorder known as IH during pregnancy may manifest in the third trimester. Small pustules or red, scaly patches grouped in a herpes-like pattern make it distinctive from any other skin condition. This condition usually improves after childbirth but may reoccur in future pregnancies. IH is capable of having severe clinical signs, including fever, chills, nausea, vomiting, and diarrhea. We encounter maternal complications rarely these days, but when they occur, they can cause delirium, seizures, and tetany due to hypocalcemia. The intrauterine fetus needs to be closely monitored for fetal anomalies, placental insufficiency, stillbirth, or intrauterine fetal demise (IUFD) [3,4]. The majority of these cases of GPPP or IH have a prior distinct history pre-existing in the family [5]; however, in a few instances, the condition arises de novo [6]. Less than 350 cases were documented in the literature in 1995, and only 10 cases were treated, according to a review of the literature [3]. This implies that there is little knowledge about how to manage and cure this condition. Usually, the rash appears in the last trimester and disappears after delivery. Although there have been links between hypocalcemia and hypoparathyroidism, a conclusive connection has not yet been made due to the small number of instances that have been recorded [3,6]. So, early diagnosis and timely care are mandatory in this context.

## Case presentation

**Figure 1:**
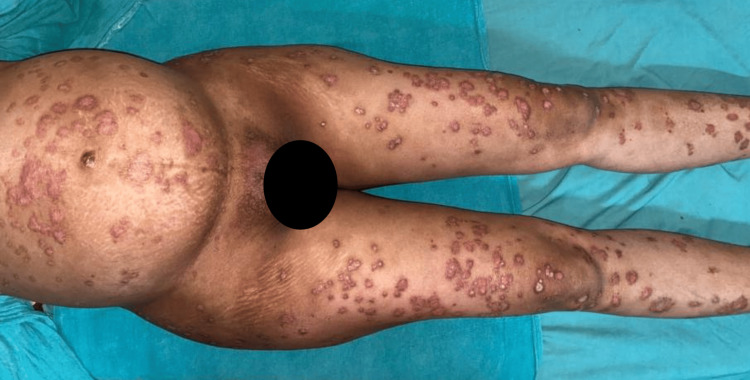
Erythematous plaques seen on distended abdomen and lower extremities.

**Figure 2:**
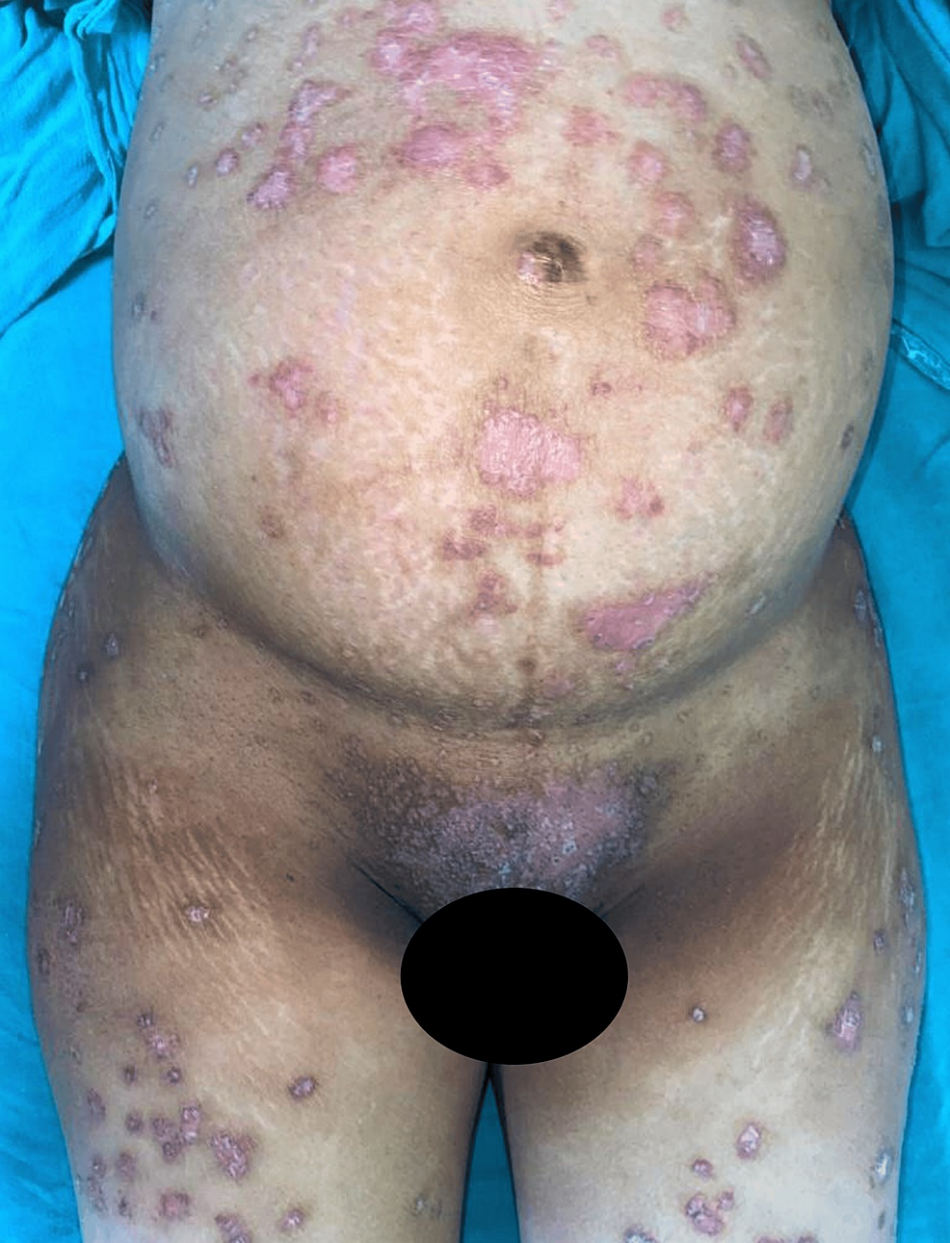
Erythematous plaques were observed on the abdomen, inguinal area, thighs, and perineal region.

A 26-year-old woman, 3rd gravida, para 1, live issue 1 (G3P1L1) with one spontaneous abortion, and at 36 weeks of gestation, presented to the ED with complaints of backache and pain in the lower abdomen for one day. She had extensive generalized erythematous-squamous plaques with polycyclic borders, intense pruritus, and was covered with pustules. At the time of presentation, skin lesions had just started to erupt a week back. There was a similar history of lesions during her first pregnancy, which spontaneously resolved in the postpartum period. She had intertriginous psoriasis for 10 years and had never seen a dermatologist for it. When her psoriasis worsened a few years back, she was hospitalized and was treated with injectable steroids, immunosuppressants, and emollients. She then discontinued the medications for relief. The patient did not take any medication during her present pregnancy and had no known personal or family history of psoriasis or any other skin conditions. There was no prior history of smoking. There was no history of indomethacin, ritodrine, or beta-blocker use during pregnancy; drugs that are commonly used in pregnancy and are implicated as risk factors for psoriasis. On examination, her temperature was 100 degrees Fahrenheit, blood pressure was 110 mmHg systolic and 78 mmHg diastolic, her heart rate was 112 beats per minute, and her respiratory rate was 14 per minute. On inspection, we noted large annular, non-tender erythematous plaques that were quite itchy and covered more than 50% of her body surface area. These plaques were covered with multiple tiny pustules with yellowish content and honey-like scales, as shown in Figures 1-2.

**Figure 3:**
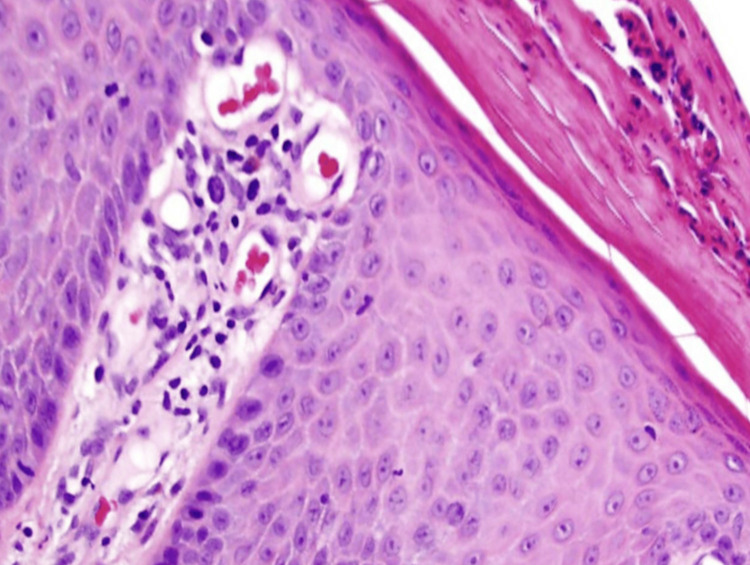
Skin biopsy section stained with H&E at 50x magnification showing parakeratosis, hyperkeratosis, elongated rete ridges, reduced stratum granulosum, and a thin suprapapillary plate.

Mucous membranes were not involved. The patient reported decreased fetal movements; hence, obstetric ultrasonography was done, which showed late-onset fetal growth restriction, with color Doppler showing high resistance flow in bilateral uterine arteries. The patient was admitted. The appearance of pustules on the erythematous-squamous base, the clinical criteria, and the presence of fever led to the preliminary diagnosis of IH with constitutional symptoms. Other diagnoses were taken into consideration. A skin biopsy was taken and sent for histopathological examination (Figure 3). It revealed retention nuclei in the Stratum corneum (known as parakeratosis), thick Stratum spinosum (known as hyperkeratosis), elongated rete ridges with reduced Stratum granulosum, and thin suprapapillary plate. These findings were consistent with IH.

However, no direct immunofluorescence (IFA) was performed. Laboratory investigations were as follows: hemoglobin was 11.2 g/dL, total leucocyte count was 24,800/ mm³, showing neutrophilia. Her renal function test and liver function test results were both within normal range. She had a 323 μ/L rise in lactate dehydrogenase (LDH) enzyme in her serum. C-reactive protein was 1.88 mg/dL (normal: <1.0 mg/dL), and the erythrocyte sedimentation rate (ESR) was 80 mm/hour. She was diagnosed with IH with constitutional symptoms in preterm labor with IUGR and uteroplacental insufficiency. The patient was started with antipyretics and analgesics (oral paracetamol 650 mg thrice a day) and prophylactic antibiotics (oral cefixime 1 mg twice a day). Due to the compromised state of the fetus, the patient was allowed to go into labor spontaneously; however, labor was not yet augmented due to acute febrile illness. She was also given supportive therapy in the form of IV fluids and psychosocial support. Prednisolone was given orally in a dosage of 0.5 mg/kg body weight (30 mg/daily) and was immediately initiated. Betamethasone, 0.1% ointment, was also prescribed for topical application twice daily. In the next 36 hours, the patient went into the active phase of labor and vaginally delivered a female child, 2.2 kg, shifted to the mother's side. There was no evidence of gross congenital anomalies. She improved in the next 3-4 days postpartum. She was then discharged on day 5 of delivery on prednisolone 30 mg/day dose for outpatient follow-up. She returned 10 days after discharge, noting improvement. Prednisolone was then prescribed with a tapering dosage: from 20mg/day down to 10mg/day over the subsequent 2 weeks. One month after delivery, the lesions were totally resolved. There were no further rebounds or relapses seen. On follow-up after six months postpartum, neither she nor the baby had developed skin conditions.

## Discussion

Pathogenesis

The reason behind IH is presently unknown. However, studies suggest that genetic factors, like family history, may contribute to its development. Specifically, the interleukin 36 receptor antagonist (IL36RN) gene, which creates the IL-36 receptor antagonist, has been discovered to have mutations in a significant number of patients with generalized pustular psoriasis who do not have a background of psoriasis vulgaris [7]. There are several cytokines involved in different types of pustular dermatoses, including tumor necrosis factor, IL-17A, and IL-22. These cytokines generate interleukin 36 (IL-36), which is not present in healthy skin. In Japan, two patients with IH had homozygous and heterozygous IL36RN mutations [8]. In China, a patient with IH had a mutated IL36RN gene. This mutation may potentially predict IH and reduce the risk to both mother and baby. However, it is not yet determined how many IL36RN-negative patients will develop IH [8]. Establishing a cause-and-effect relationship can be difficult, but some diseases, such as hypocalcemia, have been linked to IH. Hypoparathyroidism, hypoalbuminemia, low vitamin D levels, and malabsorption-related decreased ionized serum calcium concentrations are conditions that have been identified as underlying causes of hypocalcemia in IH patients [9]. One of the most common thyroid conditions associated with IH is hypoparathyroidism [9]. Although the exact causes are unknown, certain medications have been known to induce IH. For instance, in one case, IH was triggered by consuming N-butyl-scopolammonium bromide. The symptoms of IH appeared during the 34th week of pregnancy, after five days of taking the drug. Additionally, according to Kuwabara Y et al. [10], ritodrine hydrochloride, a medicine used to treat preterm uterine contractions, can also lead to IH.

Clinical presentation

The common symptoms are erythematous patches with pustules that appear in areas where skin folds. These patches may spread outward and become crusted or even infected. Occasionally, lesions resembling Pemphigus vegetans may also be present, although this is rare.

Complications

IH can cause serious complications for both the mother and the fetus. One of the most common problems is a change in serum calcium levels, which can lead to placental insufficiency and electrolyte imbalances. IH can cause systemic symptoms such as leukocytosis, increased ESR, hypocalcemia, hypoalbuminemia, and iron deficiency anemia. It can also lead to fever, chills, diarrhea, hypovolemic shock, seizures, and malaise. In a study conducted by Huang YH et al., it was found that a woman who was diagnosed with IH during her 32nd week of pregnancy experienced complications due to gestational hypertension [11]. IH is also associated with a higher risk of perinatal problems, including fetal growth restriction caused by placental insufficiency, preterm membrane rupture, and even stillbirth. There is a recorded case of a mother who exhibited typical IH symptoms in the eighth month of her pregnancy and delivered an infant diagnosed with Ondine's curse (central hypoventilation syndrome) [12]. Recurrences have been documented in up to nine pregnancies involving women under the age of 18.

Treatment options

The main treatment obstacles we encounter are the mother and fetus's critical state and the potential teratogenicity of the medications used to treat IH. Systemic corticosteroids continue to be the go-to treatment for pustular psoriasis. A total of 15-30 mg per day is the recommended starting dose for mild to moderate patients. If necessary, the dosage can be increased to 40-60 mg or even 80 mg daily. Using corticosteroids while pregnant may increase the risk of cleft palate. However, since IH usually develops in the late third trimester, using corticosteroid medication may still be safe. High-potency topical corticosteroids may limit fetal development, so it is advised to use low to moderate-potency options instead of highly potent or very-potent ones. For patients who do not respond to corticosteroids, cyclosporine is an alternative option. Studies show that cyclosporine has been used to treat 14 patients with this condition, often combined with systemic corticosteroids, with varying results [5,13]. The dose administered was between 2 and 7.5 mg/kg/day, with varying results noted. Once cyclosporine treatment begins, the dosage of corticosteroids can be gradually reduced. Although they cannot entirely cure IH, antibiotics seem to be an effective treatment for the condition. Among the antibiotics that have shown effective in treating IH include ampicillin, macrolide, and clofazimine.

Infliximab and adalimumab are anti-TNF drugs that are actually categorized as category B drugs in pregnancy. TNF-blockers do not appear to raise the risk of fetal problems in pregnant women, according to recent studies. However, the US FDA has not yet approved its routine use during pregnancy. A case of recalcitrant severe pustular psoriasis in a pregnant woman has been successfully treated with ustekinumab, according to reports. When there is an inadequate response to corticosteroids, narrow band-ultraviolet B (NB-UVB) is added to therapy because it is regarded as a safe alternative during pregnancy. The third trimester, during which IH is most prevalent, is not a major concern, even though NBUVB has been associated with reduced folate levels in pregnant women. Conversely, a folate deficiency in the first trimester can lead to neural tube defects [14]. Psoralen and ultraviolet A (PUVA) may result in low birth weight newborns even though it is generally safe and has not been associated with an increased risk of congenital abnormalities or infant mortality. Systemic retinoids have been used to treat IH postpartum, even though they are contraindicated during pregnancy because of their teratogenic effects. If considering systemic retinoid administration after delivery, it is crucial to obtain the mother's informed consent for the appropriate contraceptive method. Despite being prohibited during pregnancy, methotrexate has been used successfully to treat IH during puberty [15]. A brief review of various dermatoses of pregnancy is shown in Table 1.

**Table 1:** A review of dermatoses during pregnancy: histopathological findings, fetal risks, and treatment modalities. UVB: Ultraviolet B.

Diagnosis	Trimester	Description of rash	Histopathology	Fetal risk	Treatment
Pemphigoid gestationis	3^rd^	Vesicobullous eruption arising on urticated erythematous papules and plaques, periumbilical involvment	Subepidermal blister, positive immunofloresence	Prematurity, small for dates	Steroids, antihistamines
Polymorphic eruption of pregnancy	3^rd^	Pruritic urticarial papules arising within striae, periumbilical sparing	Not specific	No fetal risk	Steroids, antihistamines
Atopic eruption of pregnancy	1^st^-2^nd^	Eczematous or papular lesions, background history of atopic dermatitis	Not specific	No fetal risk	Steroids, antihistamines, UVB

## Conclusions

While IH seems to be a misnomer as it is not caused by viral or bacterial infections, it appears to be a variation of pustular psoriasis. It has a genetic, immunological, and biochemical backdrop that poses significant risks to both the mother and fetus. Maternal sepsis is a well-recognized complication that requires careful diagnosis and management. Patients with erythroderma need appropriate treatment, hydration, electrolyte replenishment, adequate analgesics, antibiotics for secondary infections, and consistent fetal monitoring. IH is a life-threatening dermatosis that poses risks to both the woman and the fetus. Managing IH can be challenging, and an accurate diagnosis is crucial for devising an evidence-based therapeutic strategy.
